# Curing and Degradation Kinetics of Phosphorus-Modified Eugenol-Based Epoxy Resin

**DOI:** 10.1021/acsomega.4c06532

**Published:** 2025-01-27

**Authors:** Danuta Matykiewicz, Beata Dudziec

**Affiliations:** 1 Faculty of Mechanical Engineering, Poznan University of Technology, Piotrowo 3, Poznan 61-138, Poland; 2 Faculty of Chemistry and Center for Advanced Technologies, Adam Mickiewicz University in Poznan, Uniwersytetu Poznanskiego 10, Poznan 61-614, Poland

## Abstract

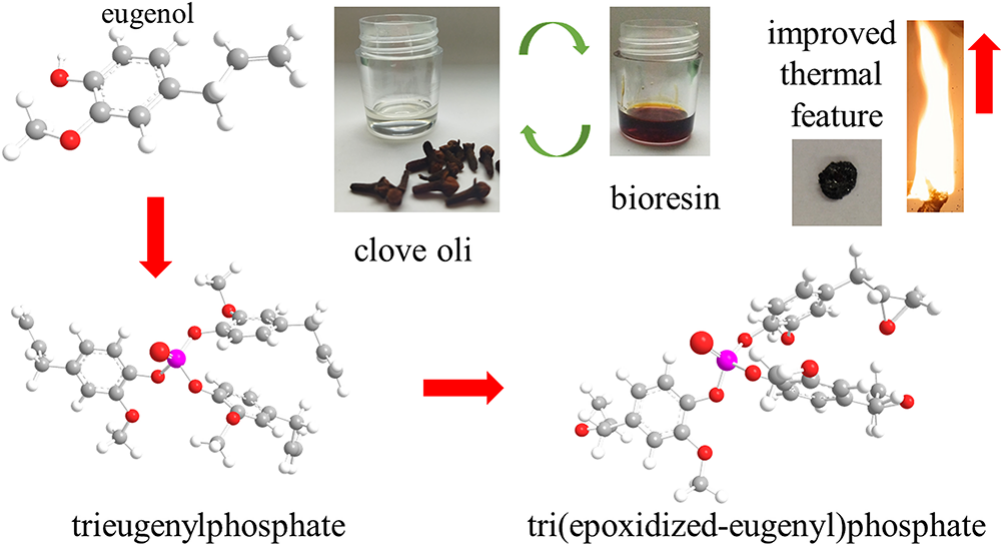

Decreasing fossil fuel resources results in a growing demand for polymeric materials obtained from renewable raw materials, such as eugenol. Therefore, this work aimed to assess the kinetics of cross-linking and degradation of epoxy resin obtained from eugenol derivatives and cured with three types of amines: aliphatic: triethylenetetramine (TETA); aromatic: diaminodiphenylmethane (DDM), and cycloaliphatic isophorone diamine (IDA). The product was characterized by ^1^H, ^13^C, and ^31^P NMR as well as ESI MS techniques. The curing kinetics of the biobased resin was studied using differential scanning calorimetry (DSC) at different heating rates. Fourier transform infrared (FTIR) spectroscopy was used to assess chemical changes in bioepoxy monomer after the curing process. The DSC method confirmed the occurrence of an exothermic curing reaction of the tested bioresin for all tested curing agents. The peak temperature *T_p_* and enthalpy Δ*H* values determined during DSC analysis depended on the type of curing agent. The highest values of *T_p_* (142.6–161.4 °C) and Δ*H* (28.5–38.3 J/g) were recorded for the TEEP + DDM composition. For the remaining compositions, the values were lower and were as follows: for TEEP + TETA, *T_p_* = 115.0 to 129.9 °C and Δ*H* = 12.4–26.5 J/g and for TEEP + IDA, *T_p_* = 118.0–137.1 °C and Δ*H* = 16.3 to 35.5 J/g. According to the Kissinger and Ozawa model, the activation energy of the resin cross-linking process was determined. The calculated activation energies according to Kissinger and Ozawa were 65.38 and 55.90 kJ/mol for TEEP + TETA, 60.09 and 63.84 kJ/mol for TEEP + DDM, and 57.36 and 60.85 kJ/mol for TEEP+IDA, respectively. The kinetics of thermal degradation of the eugenol-based resin were studied by thermogravimetric analysis (TGA) in a nitrogen atmosphere. Moreover, it should be emphasized that compared to commercial resins, bioresin has a much lower maximum degradation rate determined by DTG and a higher amount of char residue after thermal degradation, both in nitrogen and in air.

## Introduction

With the increasing ecological awareness of producers of plastic products, the demand for polymeric materials of biological origin increases. Therefore, many scientific works focus on the use of compounds of plant origin as substrates in the synthesis of biopolymers.^1,2^ Cardanol, obtained from cashew nut shell liquid, is commonly used to prepare polymers.^3,4^ Due to the industrial availability of vanillin, most often obtained from guaiacol or lignin, it is also widely used in the production of polymers such as phenolic, epoxy and benzoxazine resins, polyesters, acrylic and methacrylate polymers.^5−7^ Eugenol is a volatile phenolic component of clove oil obtained from Eugenia, grown mainly in the areas of Indonesia, India and Madagascar.^8^ Eugenol has low chemical stability and is susceptible to oxidation and other chemical reactions.^9^ The presence of a phenolic group in eugenol gives it antimicrobial activity and the ability to scavenge reactive oxygen species.^10^ Therefore, it is mainly used in cosmetics but also for the production of polymeric materials.^11,12^ Watanabe et al. manufactured coating materials using urushiol analogues that were synthesized via a simple three-step route from eugenol (4-allyl-2-methoxyphenol), an allyl-substituted guaiacol.^13^ Biobased films were obtained on various substrates by spin-coating a solution of the urushiol analogues and iron(II) acetate. In turn, novel eugenol derivatives with acrylic moieties for orthopedic and dental cements application were described by Rojo et al.^14^ Consiglio et al. described a novel method of producing methacrylate polymers containing phenolic groups based on eugenol and their effective polymerization with 2-hydroxyethyl methacrylate.^10^

A derivative of triglycidyleugenol (3EPOEU) was used to prepare amine-cured epoxy materials as an alternative to standard materials based on diglycidylether of bisphenol A (DGEBA).^12^ 3EPOEU thermosets showed good thermal stability, higher Tg and mechanical properties in comparison to DGEBA. A new type of biobased bisepoxide 2,2′-diglycidyl ether-3, 3′-dimethoxy-5,5′-diallydiphenylmethane (BEF-EP) was synthesized from eugenol and its curing agent 3-methoxy-4-hydroxy-phenylbenzimidazole (VBZMI) from and vanillin by Jiang et. all.^15^ Resin composition with a new type of curing agent showed higher thermal stability, a higher glass transition temperature and a higher contact angle than resin composition cured with benzimidazole BZMI. Chen et al. described the process of synthesizing fully biobased epoxy compounds from eugenol by synesis fully biobased epoxy compounds from eugenol through the esterification of the phenolic group, followed by the oxidation of the allylic group.^16^ Ecochard et al. presented a method for the synthesis of four biobased epoxy monomers from cardanol and eugenol.^17^ The high aromatic density of eugenol-based materials allows for obtaining materials with a high glass transition temperature, while the long aliphatic chains of cardanol monomers lead to flexible thermosetting materials with a low Tg. Liu et al. described biobased eugenol epoxy cured with different epoxy-anhydride stoichiometric ratios.^18^ The curing kinetics and properties of the epoxy resin system with eugenol biobased reactive epoxy diluent were evaluated by Chen et al.^19^ Celikbag et. al proved the self-curing phenomena of bio-oil-based epoxy resin to be produced by hydrothermal liquefaction of loblolly pine.^20^ Thermal stability and properly selected hardening parameters of biological resins are key information enabling their application on a larger scale.^21^ Therefore, many studies focus on determining the kinetics of curing and degradation of epoxy materials.^22,23^ Renewable natural resources, such as vegetable oils, saccharides, polyphenols and phenols, are successfully used to produce bio resins.^24,25^ Phosphorus-modified biobased compounds have recently been evaluated for their use as flame retardants.^26,27^ Liu et all described, flame retardant eugenol-based thiol–ene polymer networks.^28^ Zhou et al. used eugenol-based flame-retardant epoxy monomer in combination with commercial bisphenol A-type epoxy resin to produce an epoxy resin with improved flame retardancy.^29^ However, the use of these substrates for the synthesis of an epoxy resin, which itself has better thermal properties, and the evaluation of its cross-linking have not yet been thoroughly characterized.

The intrinsic brittleness and flammability of epoxy resins remain significant drawbacks. To overcome these limitations, recent research has focused on enhancing epoxy resins with natural compounds and flame retardants. The type of curing agent used has a significant influence on the final properties of the cured resin and the selection of its processing methods. The group of alkaline curing reacting with epoxy resins based on polyaddition includes amines containing at least two active hydrogen atoms in the molecule. These include, among others, diamines, aliphatic polyamines, aromatic and cycloaliphatic amines. Their broader or narrower application is determined mainly by technical requirements such as curing temperature and time, properties of the cured material and price. Cross-linking using polyamines such as diethylenetriamine and triethylenetetramine most often occurs at room temperature, and the cured material is characterized by high mechanical strength and chemical resistance.^30^ These are low-viscosity liquids used where it is technically impossible to apply an elevated temperature during curing. However, they are sensitive to ambient moisture which can cause deactivation of the curing agent. The reaction rate is high, as is the amount of heat released during the process. For this system, subsequent curing at an elevated temperature can be used to increase the heat resistance of the materials. Cycloaliphatic amines, in comparison to aliphatic ones, are less sensitive to moisture during hardening. Some of them, e.g. isophorone diamine, are used for the production of coating materials, casting compositions with excellent heat resistance and mechanical strength.^31^ In turn, aromatic amines have a nitrogen atom directly bonded to a carbon atom in the aromatic ring. The most commonly used aromatic amines include 4,4' -Diaminodiphenylsulfone (DDS) and 4,4'-Diaminodiphenylmethane (DDM).^32^ However, they require curing at elevated temperatures, and epoxy materials containing them are characterized by much better thermal properties than epoxy materials cured with aliphatic amines. Epoxy compositions with DDM are used for casting electrical parts, and production of laminates and adhesives even in high humidity conditions.

Eugenol, a biobased phenolic compound derived from clove oil, offers notable benefits such as increased flexibility and natural antimicrobial properties. Phosphorus compounds are widely recognized for their superior flame-retardant capabilities. Research on the use of eugenol and its isomers for the synthesis of epoxidized precursors is bringing more and more favorable results that can be used on a larger scale in the future.^33^ Triethylenetetramine (TETA), due to its low viscosity, is recommended for the production of laminates, while isophorone diamine (IDA), with an even lower viscosity, can be used as an efficient diluent for epoxy systems, which ensures better saturation of reinforcing fibers.^34^ 4,4′-Diaminodiphenylmethane occurs in solid form but usually guarantees a high glass transition temperature (*T*_g_) of hardened materials.^35^ The use of three types of curing agents was aimed at verifying their reactivity with a new type of bioresin. This will help determine the area of its future application. The TETA and IDA hardeners are intended for hardening at ambient temperature, while DDM is intended for hardening at elevated temperatures. The disadvantages of cross-linking agents based on low molecular weight linear polyamines such as triethylenetetramine include their high hygroscopicity and vapor pressure, tendency to bind carbon dioxide from the air and often too high reactivity. However, the extension of the polyamine hardener chain causes a decrease in reactivity and vapor pressure, but an increase in its viscosity, which is a very unfavorable phenomenon during processing.^36^ The graphical abstract presents the main assumptions of the work, including the use of eugenol obtained from clove oil as a raw material for the production of epoxy resin and the strong synergistic effects of the integration of eugenol with a phosphorus compound to improve the thermal properties of epoxy resins. Therefore, this work aimed to assess the kinetics of cross-linking and degradation of epoxy resin obtained from eugenol derivatives and cured with three types of amines: triethylenotetraamine (TETA), diaminodiphenylmethane (DDM) and isophorone diamine (IDA). The novelty of the described research includes previously undescribed characteristics of the curing and degradation process of resin produced based on raw materials from sustainable sources.

## Experimental Section

### Materials

Eugenol for synthesis 99%, phosphorus(V) oxychloride 99%, 3-chloroperbenzoic acid ≤77% (m-CPBA), triethylamine ≥99.5%, ethyl acetate ≥99.5%, sodium bicarbonate ≥99.7%, anhydrous sodium sulfate 99%, 4,4′-Diaminodiphenylmethane ≥99.7% (DDM), were obtained from Sigma-Aldrich (Poland); triethylenetetramine (TETA), isophorone diamine (IDA) were purchased from (CIECH Sarzyna S.A., Poland). For comparison of thermal properties (TGA), samples were also prepared from commercial resins based on DGEBA (Epidian 5 produced by CIECH Sarzyna S.A., Poland) and 50% biosourced epoxy resin GEDEO (Crystal Resin Bio PRO produced by Pebeo).

### Synthetic Route of Tris(4-allyl-2-methoxyphenyl) Phosphate (TEP)

**Figure 1:**
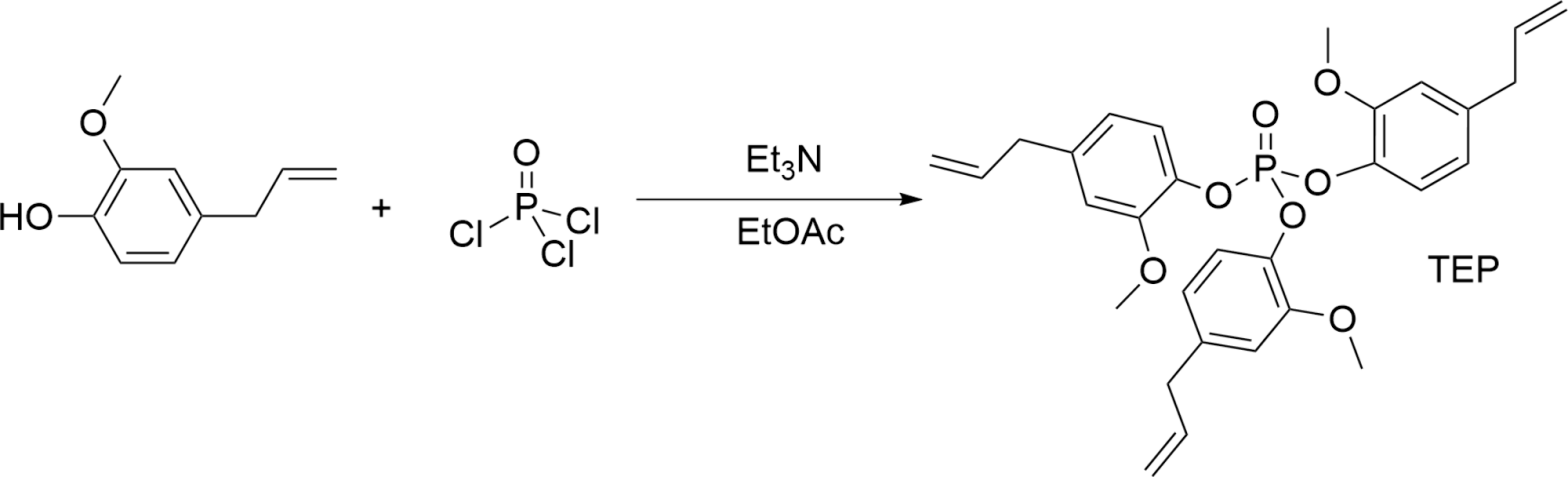
Synthesis of tris(4-allyl-2-methoxyphenyl) phosphate (TEP).

The compound was obtained by the reaction according to Faye et al.^37^ (following reaction path presented in Figure 1): 10 g of eugenol was dissolved in 100 mL of ethyl acetate and triethylamine was added, then 3.12 g of phosphorus(V) oxychloride was added dropwise to the solution, maintaining the temperature at 0–5 °C for 30 min. Next, the reaction solution was stirred at room temperature for 16 h. After the reaction was completed, the mixture was diluted in 100 mL of ethyl acetate and washed three times with 100 mL of deionized water and once in 100 mL of saline solution. The organic layer thus obtained was dried over anhydrous sodium sulfate and filtered. The filtrate was distilled under reduced pressure to obtain 10 g of TEP as a yellow oily liquid (78% yield). The product was characterized by ^1^H, ^13^C, and ^31^P NMR as well as ESI MS techniques.

^1^H NMR (400 MHz, CDCl_3_): δ (ppm) 7.27 (3H, m, Ph); 6.65–6.81 (6H, m, Ph); 5.85–5.94 (3H −CH = CH_2_); 5.02–5.05 (3H, m, CH = CH−); 3.71 (9H, s, OCH3); 3.29–3.34 (6H, m, CH_2_–CH = CH_2_). ^13^C NMR (100.65 MHz, CDCl_3_): δ (ppm) 150.57 (C_Ph_–OCH_3_); 138.49 (CH = CH_2_); 137.95 (C_Ph_–O–P); 137.16 (C_Ph_–CH_2_); 121.32 (C_Ph_); 120.50 (C_Ph_); 116.08 (CH_2_=CH); 113.17 (C_Ph_); 55.94 (OCH_3_); 39.98 (CH_2_–CH = CH_2_). ^31^P NMR (162 MHz, CDCl_3_): – 15.82 (O = PO_3_). High-resolution mass spectrometry (HRMS) (positive-ion electrospray ionization (ESI+)) *m*/*z* calculated for [C_30_H_33_O_7_P+H]^+^ 537.2042, found 537.2039. Obtained results are in accordance with the literature.^37^

### Synthetic Route of Tris(2-methoxy-4-(2,3-epoxypropyl)phenyl phosphate) (TEEP)

**Figure 2:**
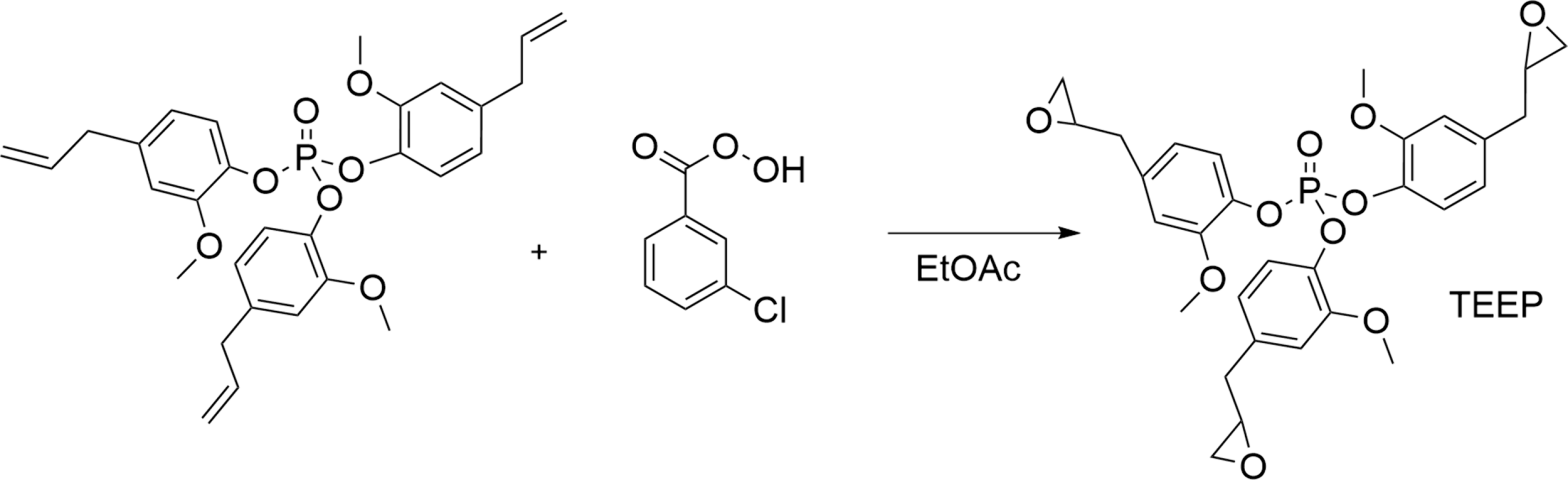
Synthesis of tri(epoxidized -eugenyl)phosphate (TEEP).

The biobased epoxy monomer was obtained by the reaction according to Faye et al.^37^ (following the reaction path presented in Figure 2). First, a cooled suspension of meta-chloroperbenzoic acid (m-CPBA, 77%) (14 g, 62.7 mmol) in ethyl acetate (200 mL) was introduced dropwise to a solution of TEP (10.2 g, 19 mmol) in ethyl acetate (50 mL). The mixture was then stirred for 30 min at 0–5 °C, then at ambient temperature for 24 h. The solvent was then evaporated and the excess m-CPBA and *m*-chlorobenzoic acid was neutralized with saturated sodium bicarbonate solution and extracted with ethyl acetate. The organic layer was washed several times with saturated sodium bicarbonate solution, brine and deionized water. Then, the organic layer was dried over anhydrous sodium sulfate and filtered. The filtrate was distilled under reduced pressure to give 10.3 g of TEEP as a brown viscous oil (78 yield). The product was characterized by ^1^H, ^13^C, and ^31^P NMR as well as ESI MS techniques.

^1^H NMR (300 MHz, CDCl_3_): δ (ppm) 7.32 (3H, m Ph); 6.68–6.86 (6H, Ph); 3.76 (9H, OCH_3_); 3.12 (3H, epoxy CH–O); 2.86–2.75 (9H, epoxy CH_2_–O, CH_2_–CH–O); 2.52 (3H, epoxy CH_2_–O). ^13^C NMR (75.48 MHz, CDCl_3_): δ (ppm) 151.40 (C_Ph_–OCH_3_); 138.13 (C_Ph_–O–P); 136.79 (C_Ph_–CH_2_); 121.00 (C_Ph_); 120.60 (C_Ph_); 113.67 (C_Ph_-OCH_3_); 55.99 (OCH_3_); 52.57 (C_epoxy_), 46.76 (C_epoxy-_CH_2_), 38.23 (CH_2_–CH–O). ^31^P NMR (162.02 MHz, CDCl_3_): – 15.92 (O = PO_3_). High-resolution mass spectrometry (HRMS) (positive-ion electrospray ionization (ESI+)) *m*/*z* calculated for [C_30_H_33_O_10_P+H]^+^ 585.1890 found 585.1887. Obtained results are in accordance with the literature data.^37^

### Epoxy Curing Process

**Figure 3:**
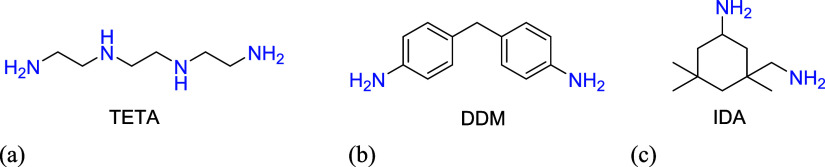
Chemical structures of curing agents (a) triethylenetetramine (TETA), (b) 4,4′-diaminodiphenylmethane (DDM), (c) 3-(aminomethyl)-3,5,5-trimethylcyclohexane-1-amine (isophorone diamine (IDA)).

The synthesized biological resin was cured using three types of amine curing agents: triethylenetetramine (TETA), diaminodiphenylmethane (DDM), and isophorone diamine (IDA). Triethylenetetramine is a cheap and widely used aliphatic amine hardener containing six active hydrogen atoms, reactive at room temperature. It is a strongly basic liquid that imparts favorable mechanical properties and weathering resistance to the cured castings. TETA possesses two primary and two secondary amine groups. Isophorone diamine (IDA) is a low-viscosity liquid and is a widely used industrial cycloaliphatic amine with four active hydrogen atoms. It is also reactive at room temperature and contains one primary aliphatic amino group and one amino group bound to a cycloaliphatic ring. Diaminodiphenylmethane (DDM) is an aromatic amine in solid form. The cross-linking reaction involving DDM is highly exothermic, so it is recommended that the initial curing phase takes place at a lower temperature, followed by an increased temperature after gelation. DDM has two primary amine groups. The structural formulas of the compounds used are shown in Figure 3.

The curing agent and the resin were stirred mechanically and then the composition was cast in Teflon mold and cured for 24 h at ambient temperature and then postcured at 150 °C for 2 h. In the case of the DDM curing agent, which melts above a temperature of 90 °C, it was introduced into hot resin (100 °C) and mixed mechanically.

The appropriate amount of amine curing agent (m_a_) was calculated based on eq 1 using the following values: the amine hydrogen equivalent weight (AHEW) and the epoxy equivalent weight (EEW). All compositions are summarized in Table 1.
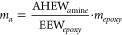
1

**Table 1:** Composition of the Tested Epoxy Systems

**Name**	**M**_**epoxy**_(g/mol)	**M**_**amine**_(g/mol)	**AHEW (g per eq.)**	**EEW (g per eq.)**	**m**_**epoxy**_**(g)**	**m**_**a**_**(g)**
TEEP+TETA	584.18	146.23	24.4	194.7	4	0.50
**TEEP+DDM**	584.18	198.26	49.5	194.7	4	1.02
**TEEP+IDA**	584.18	170.30	42.5	194.7	4	0.87

### Characterization

Proton, carbon and phosphorus nuclear magnetic resonance (^1^H, ^13^C, ^31^P NMR) analyses were carried out in deuterated chloroform (CDCl_3_) using a spectrometer at 25 °C.

Fourier transform infrared spectroscopy (FTIR) was carried out using the Jasco (Japan) FT/IR-4600 apparatus in attenuated total reflectance(ATR) mode with 64 scans at a resolution of 4 cm^–1^ in the wavenumber range of 4000–400 cm^–1^.

The cross-linking process of the resin monomer was assessed by differential scanning calorimetry (DSC) using Phoenix DSC 204 F1 Netzsch. Samples weighing approximately 10 mg enclosed in an aluminum crucible were heated from 25 to 180 °C in a nitrogen atmosphere at a heating rate of 10, 15, 20, or 25 °C/min. DSC analyses were performed immediately after completing the ingredient mixing procedure. As a result, DSC curves of the hardening process were recorded.

The thermal properties of the composites were assessed using the thermogravimetric method (TGA) in a Netzsch TG 209 F1 apparatus in the temperature range from 30 to 900 °C and a heating rate of 10 °C/min in a nitrogen atmosphere. Samples weighing 10 mg were tested in ceramic crucibles. The following data were obtained: the temperature at which the mass loss was 10% (T10%), the residual mass at 900 °C (W%) and the maximum thermal degradation temperatures from the derivative thermogravimetric curve (DTG). Moreover, according to the Kissinger method, TGA analysis was performed in a nitrogen atmosphere with a heating rate of 5, 10, 15, and 20 °C/min.

## Results and Discussion

### Fourier Transform Infrared Spectroscopy

**Figure 4:**
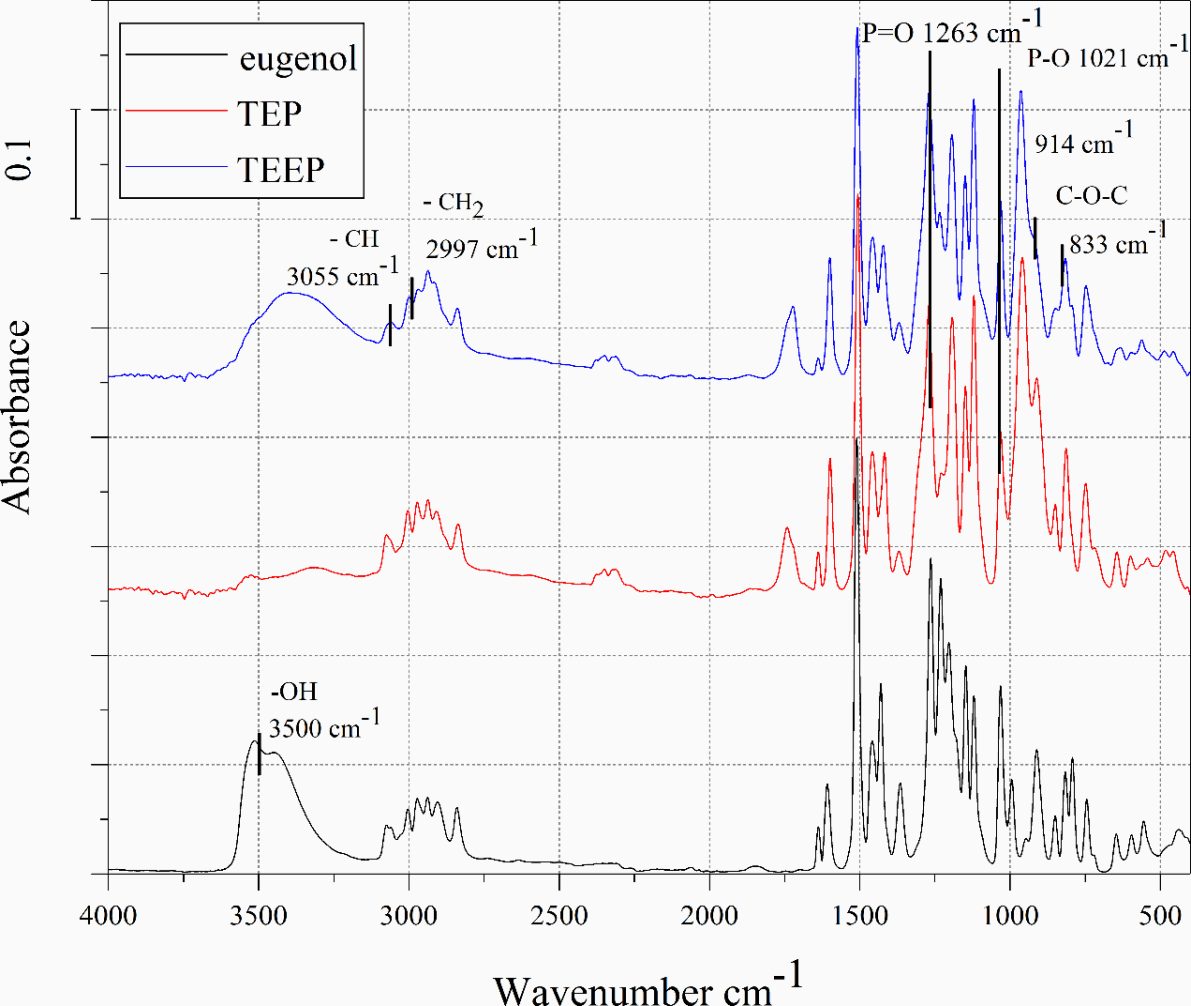
FTIR spectra of eugenol, TEP and TEEP.

FTIR spectroscopy provides detailed information about changes in the structure of a substance and enables the assessment of the effectiveness of substitution, epoxidation and curing reactions.^38^Figure 4 shows FTIR curves for eugenol TEP and TEEP compounds obtained in the synthesis. The following characteristic peaks can be observed on the FTIR curve for eugenol: in the range of 720–1250 cm^–1^ from the *v*C = C bond, the sharp peaks visible at 1600 and 1500 cm^–1^ attributed to the stretching of C = C of the aromatic group, peaks at a wavelength in the range 2700–2900 cm^–1^ can be assigned from CH_3_ groups and peak at 3500 cm^–1^ comes from hydroxyl groups.

For the TEP sample, the disappearance of the characteristic band for the hydroxyl group was observed at a wavelength of 3500 cm^–1^, which confirms the effective substitution reaction of eugenol. In addition, signals from P = O and P–O bonds were recorded at wavelengths 1263 and 1021 cm^–1^. For the TEEP sample, the sharp peaks at 914 and 833 cm^–1^ are related to asymmetric and symmetric deformation of the oxirane ring (C–O–C). The decrease in intensity of the peak at 1637 cm^–1^ comes from allyl C = C stretch.^39^ Moreover, in the TEEP spectrum, the peak at 3055 cm^–1^ may come from tension of the – CH group of the epoxide ring and at 2997 cm^–1^ from stretching of the – CH_2_ of the epoxide ring.^40^ The applied filtration and purification methods of the obtained substances brought a favorable effect; no peaks originating from the included substances were observed in the presented FTIR spectra.

**Figure 5:**
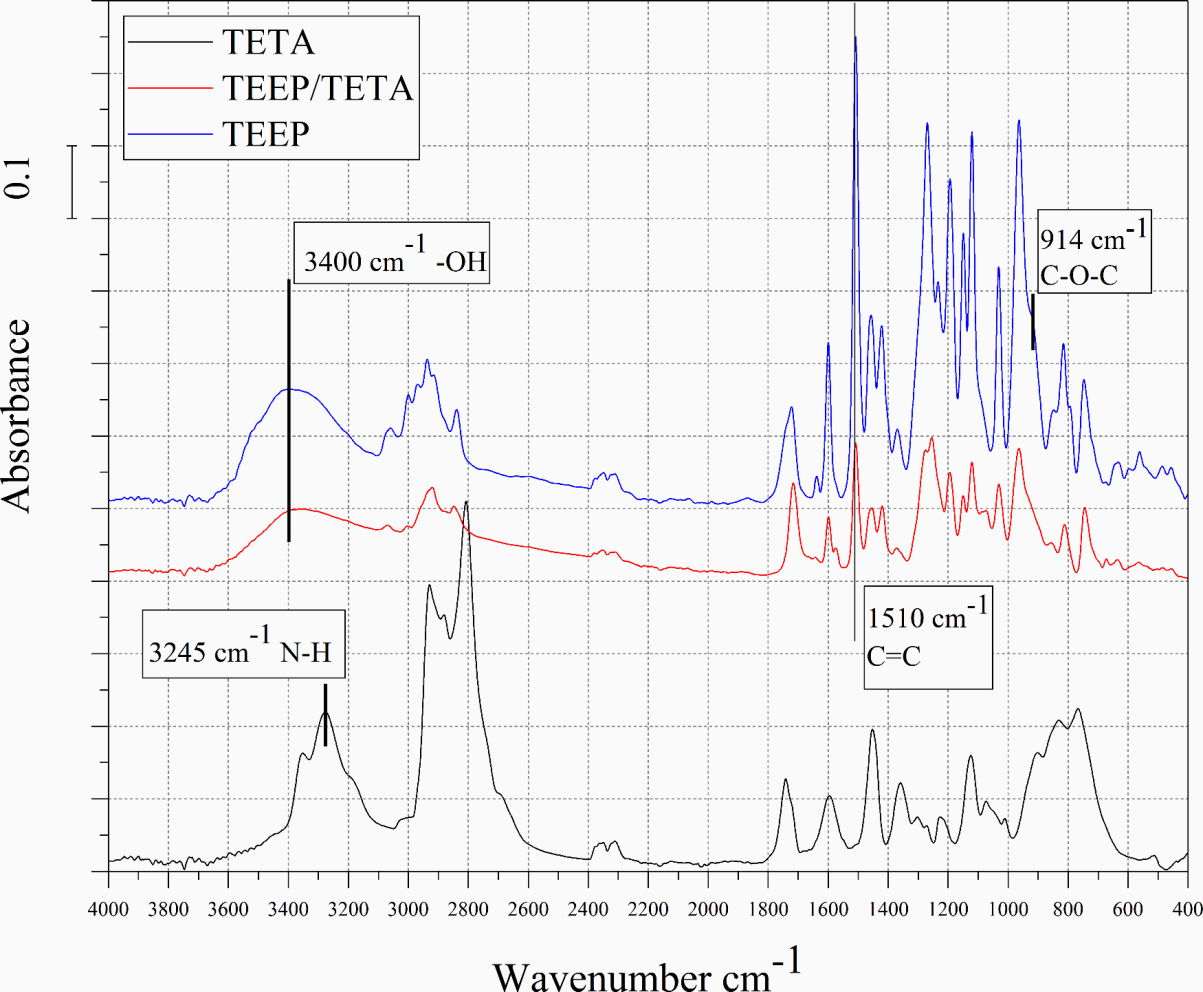
FTIR spectra of curing agent TETA and cured epoxy TEEP/TETA.

**Figure 6:**
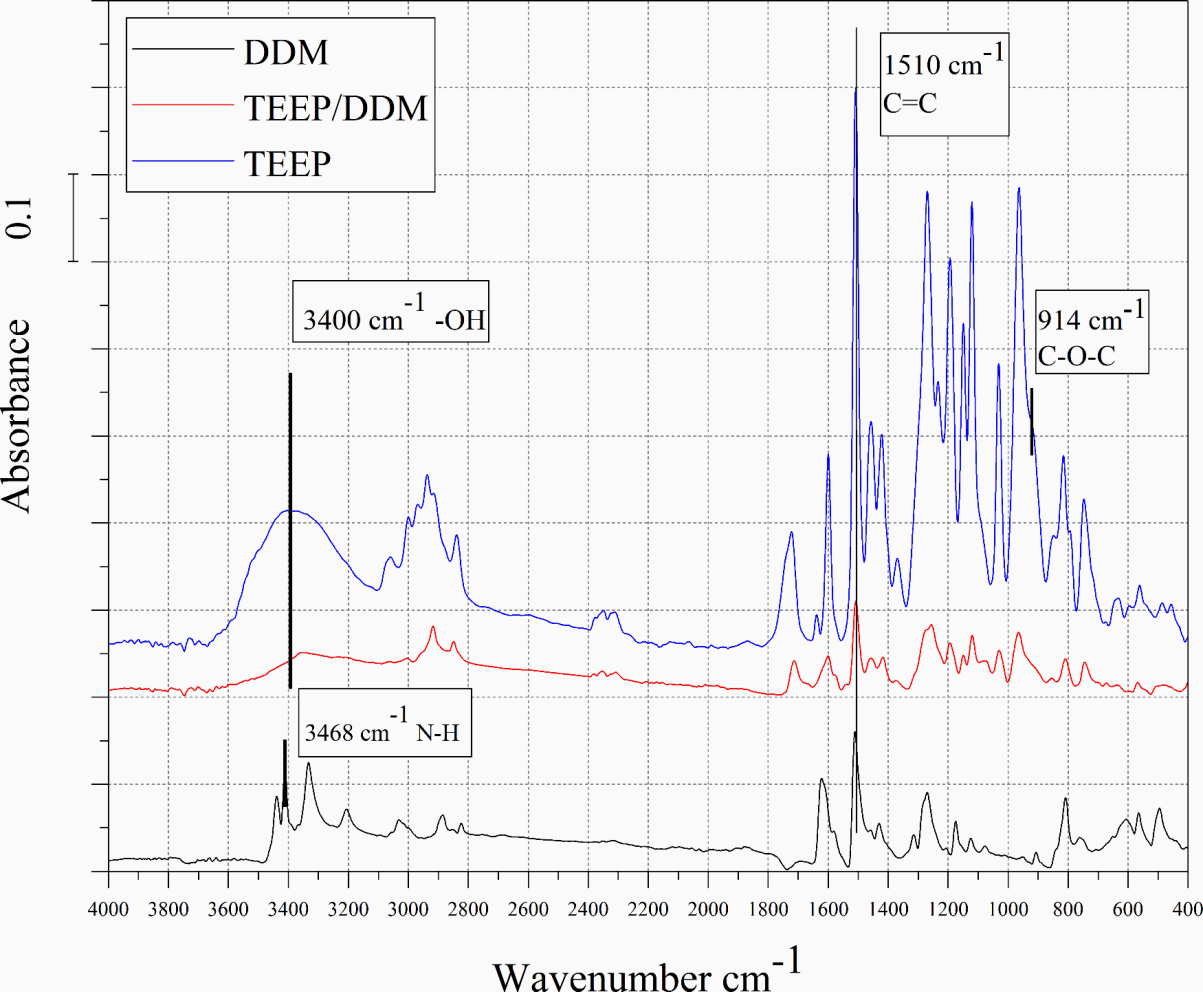
FTIR spectra of curing agent DDM and cured epoxy TEEP/DDM.

**Figure 7:**
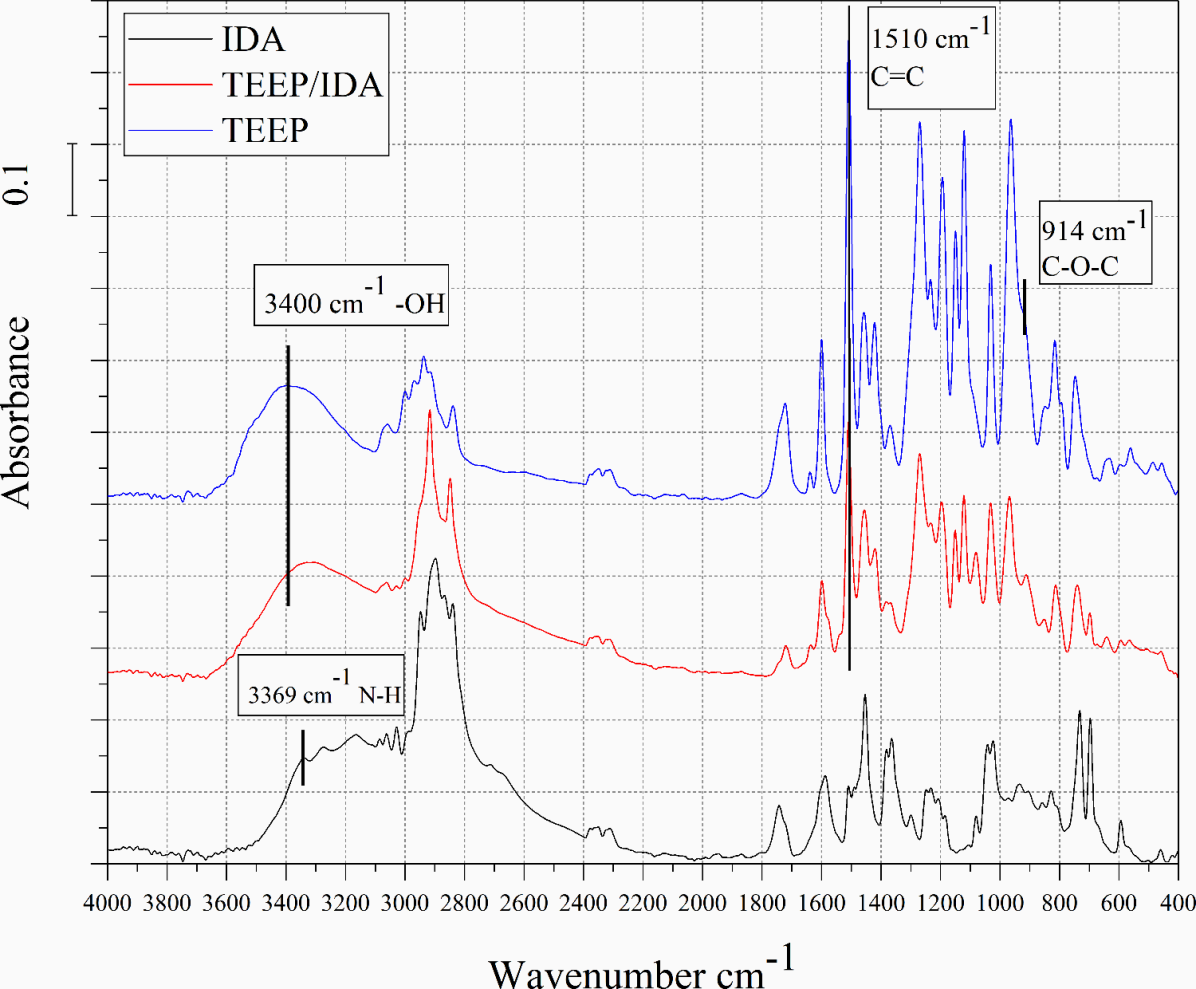
FTIR spectra of curing agent IDA and cured epoxy TEEP/IDA.

Figures 5–7 show FTIR curves for the amine curing agents used and for the cured samples. In the wavelength range 3200–3400 cm^–1^ the N–H and O–H stretching peaks can be observed.^41^ In the range 1510–1615 cm^–1^, C = C aromatic stretching peaks are visible. The peaks at 1263 and 1021 cm^–1^ P = O and P–O bonds respectively.

The amine polyaddition reaction is complex. In the first stage, the amine reacts with the epoxy group, leading to the formation of a hydroxyl group. The mechanism of the epoxy group reaction with both aliphatic and aromatic amines is similar, i.e.: after the epoxy ring opens, the amine is added, resulting in the simultaneous formation of a hydroxyl group.^42^ The adduct formed contains secondary amine groups, which can further react with additional epoxy groups.^43^ Secondary amines react with epoxy groups in a manner similar to primary amines, but the reaction proceeds at a slower rate. The final amine-cured resin may contain tertiary amine groups as well as incompletely reacted secondary amine groups.

After curing monomer TEEP with TETA, DDM, and IDA hardeners, the cured samples showed a broad band at 3200–3600 cm^–1^, attributed to −OH stretching. The occurrence of this broad band for all systems suggests ring opening of the epoxy groups in the TEEP monomer. Also, the disappearance of the characteristic amine band at 3245 cm^–1^, 3369 cm^–1^ 3468 cm^–1^ for the cured samples was visible.^40^ Furthermore, the peaks corresponding to the epoxy ring are 914 and 833 cm^–1^ disappeared after the curing reaction. It can be concluded that the spectrophotometric analysis allowed us to confirm that the materials were cured using all three types of amine-curing agents. Moreover, the ring-opening reaction between epoxy and amine groups was confirmed by recorded characteristic exothermic peaks during DSC analysis.

### Cure Kinetic Study

Control and understanding the mechanisms of the hardening process of reactive resins is important for the production process of finished products with specific functional properties or functionalities.^44^ The Ozawa equation^45,46^ and the Kissinger equation^47^ were used to evaluate the curing process of biologically based epoxy systems.^48^ The curing kinetics of the composition was assessed using the Kissinger method, according to equation 2. In this method, the activation energy is obtained by plotting ln(β/T_p_^2^) to 1/T_p_.
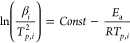
2Where β is the heating rate, T_p_ is the peak temperature on the DSC curves, R is the gas constant and *E*_a_ is the activation energy. The abbreviation *i* denotes different heating rates.

Ozawa’s method is based on the logarithm of the heating rate and the inverse of the exothermic peak temperature, i.e., ln(β) versus , according to eq (3–5). Activation energy can be appointed from the resultant slope of the linear fit. Factor A is obtained by calculating the slope of the linear fit and the y-intercept, respectively.
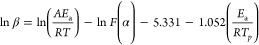
3
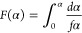
4
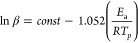
5

**Figure 8:**
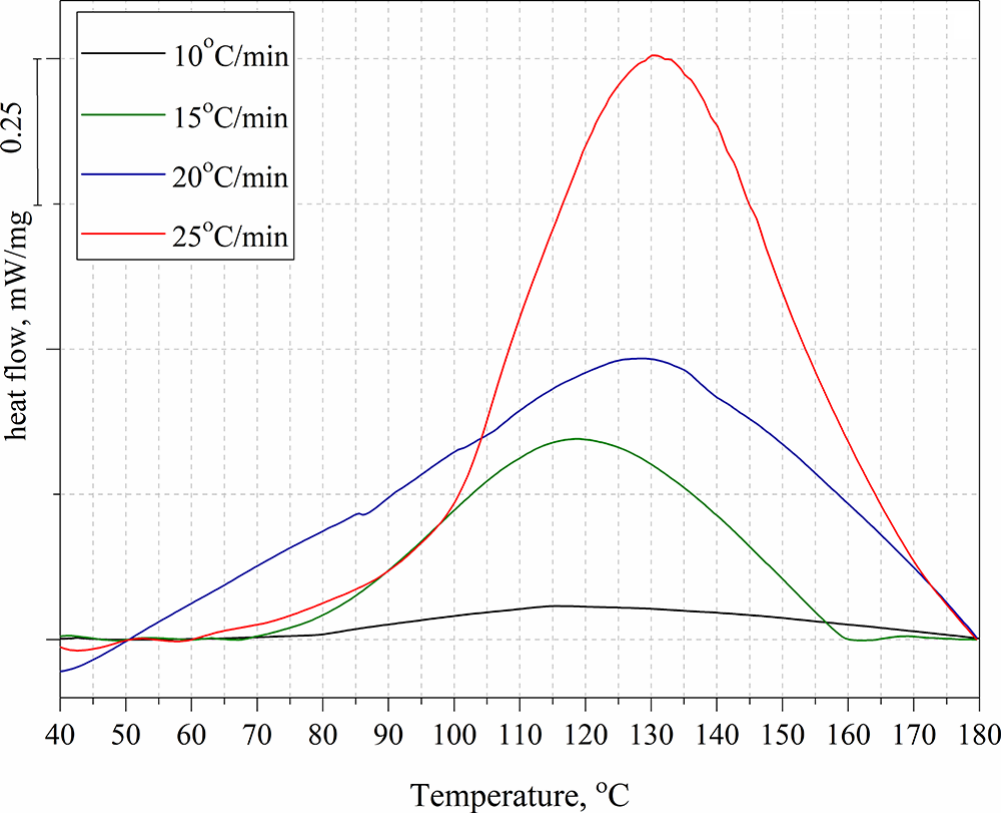
Nonisothermal DSC curves of the TEEP with TETA at different heating rates.

**Figure 9:**
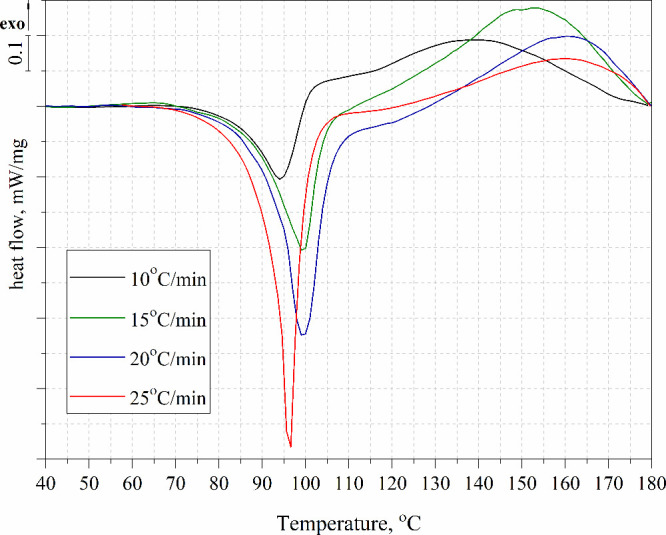
Nonisothermal DSC curves of the TEEP with DDM at different heating rates.

**Figure 10:**
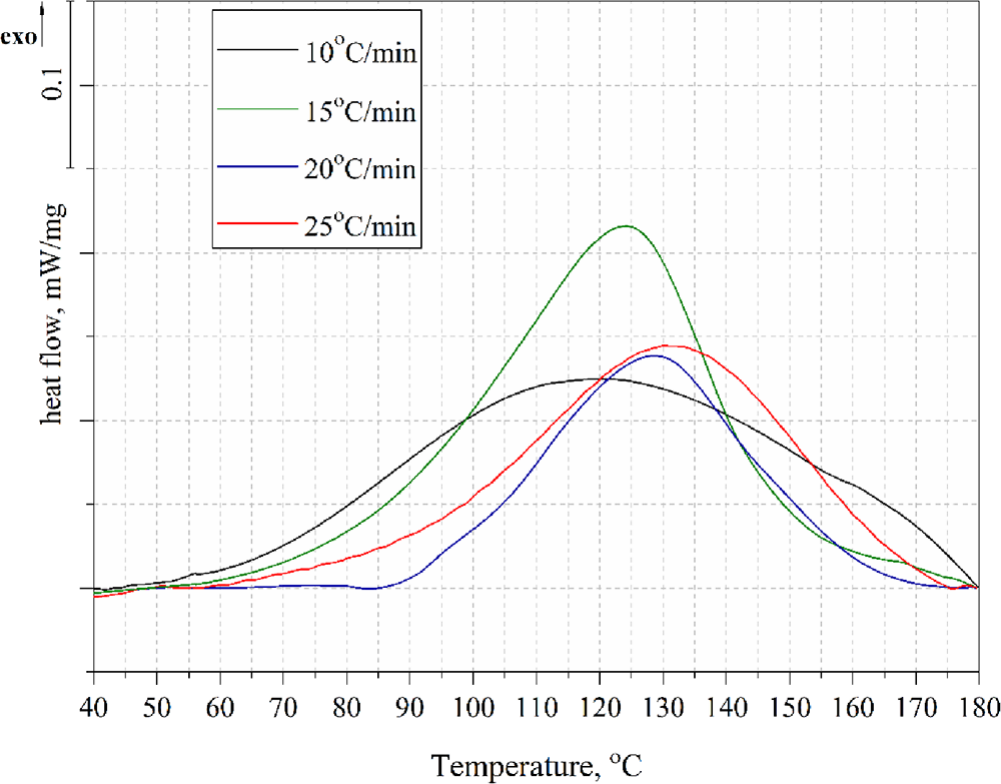
Nonisothermal DSC curves of the TEEP with IDA at different heating rates.

The course of the exothermic cross-linking reaction of the TEEP epoxy monomer depended on the type of curing agent used. The following types of compounds were selected to harden the TEEP monomer: aliphatic amine (TETA) with four amino groups, aromatic amine (DDM) with two amino groups and cycloaliphatic amines (IDA) with two amino groups. DSC data of the tested compositions from nonisothermal scans are presented in Table 2. Both TETA and IDA are in a liquid state, which makes them much easier to mix with an epoxy monomer. DDM is in the form of white crystals with a melting point in the range of 90–92 °C. The onset temperature (T_onset_) of the exothermic reaction, the peak temperature (T_p_) and the value of the exothermic effect (Δ*H*) were determined. As the heating rate increases, the T_p_ value increases. However, at higher heating rates (β= 20 and 25 °C/min) this increase was not significant. The recorded DSC curves for the tested systems are shown in Figures 8–10.

**Table 2:** DSC Data from Non-Isothermal Scans for the Kissinger and Ozawa Methods

Name	β (°C/min)	T_onset_ (°C)	T_p_ (°C)	Δ*H* (J/g)	Kissinger *E*_a_ (kJ/mol)	Ozawa *E*_a_ (kJ/mol)
TEEP+TETA	10	78.3	115.0	12.4	65.38	55.90
	15	79.1	118.2	18.1		
	20	80.4	128.9	26.5		
	25	87.0	129.9	19.2		
TEEP+DDM	10	101.0	142.6	30.7	60.09	63.84
	15	119.8	153.8	38.3		
	20	110.5	160.3	26.8		
	25	114.8	161.4	18.7		
TEEP+IDA	10	93.2	118.1	35.5	57.36	60.85
	15	92.9	124.6	25.5		
	20	99.8	131.1	16.3		
	25	92.6	137.1	17.4		

A characteristic exothermic peak was observed for all tested compositions, confirming the occurrence of the curing reaction. The peak temperature values depended on the type of hardener used as well as the heating rate. The lowest T_p_ values were recorded for the resin cured with triethylenetetramine (115–130 °C), and the highest T_p_ (142–160 °C) values for the resin cured with 4,4′-diaminodiphenylmethane. This is mainly because DDM melts first when heated, which is visible as an endothermic peak in the range of 90–100 °C. TETA is in liquid form and the reaction can occur at a lower temperature. For the TEEP+IDA system, T_p_ ranged from 118 to 137 °C. In addition, low-viscosity isophorone diamine can be used as a diluent for epoxy systems^34^

Epoxy cross-linking reactions are exothermic, therefore the reaction enthalpy values are given in the Table 2. It can be seen that the greatest heat (for = 10 °C) is released during hardening for the TEEP+IDA (35.5 J/g) and TEEP+DDM (30.7 J/g) compositions. In order to lower the process temperature, it is recommended to use curing agents containing electrophilic groups.^49^ The reactivity of epoxide groups with nucleophilic or electrophilic compounds is explained by the release of the ring strain inherent to the three-membered oxirane group. These types of curing agents can be catalysts such as Lewis acids and tertiary amines, or coreactants such as primary amines, thiols, carboxylic acids or acid anhydrides. Curing agents used as catalysts initiate the homopolymerization of oxirane rings, and the properties of the cured materials depend primarily on the chemical structure of the epoxy resin. In contrast, coreactants such as the amines TETA, IDA and DDM used contain mobile hydrogen atoms and become an integral part of the final macromolecular network. Then the chemical and physical properties of the epioxy resin are equally dependent on the epoxy monomer and the coreactant.^50^

**Figure 11:**
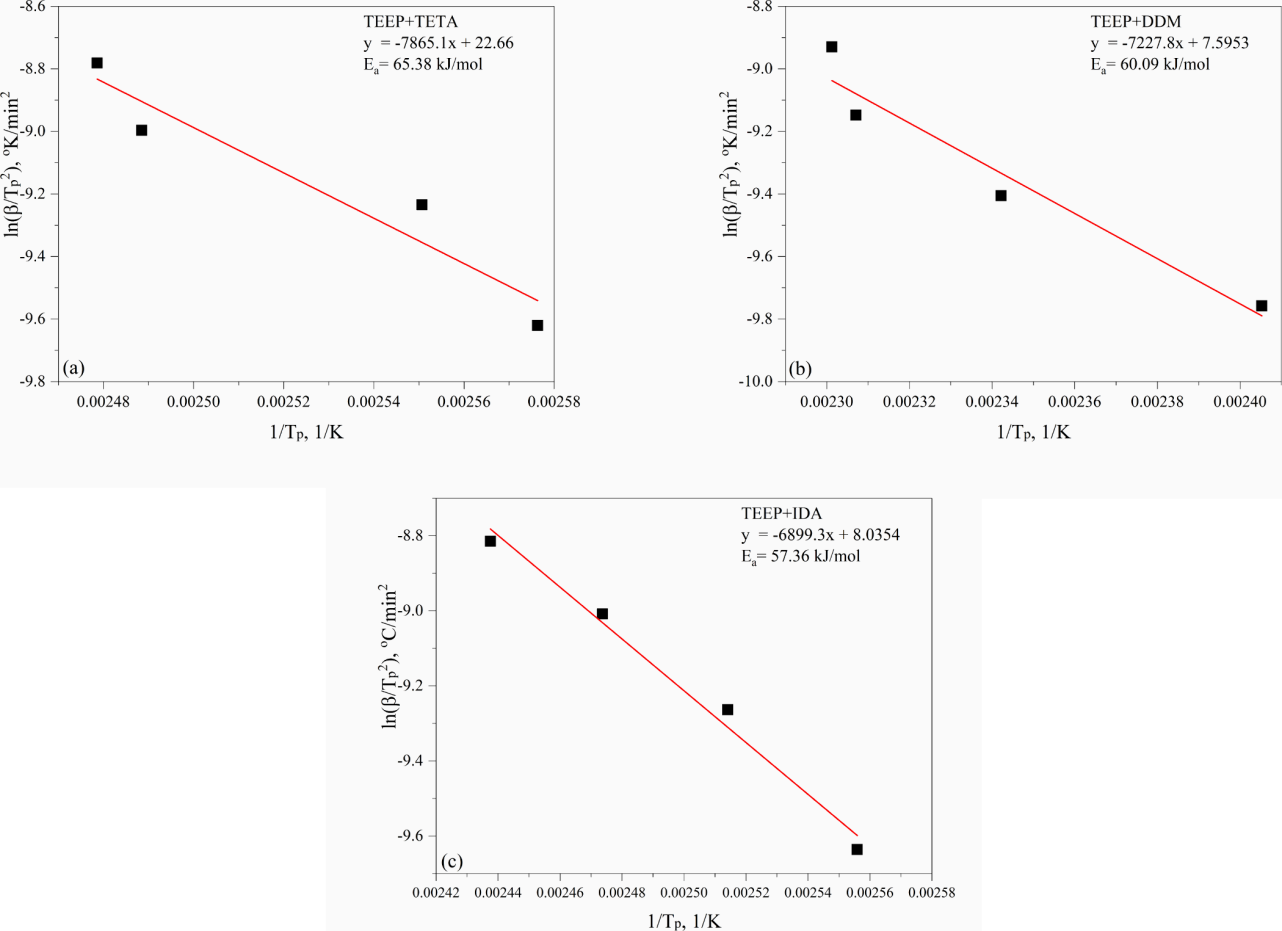
Plots of Kissinger evaluation for (a)TEEP+TETA (b) TEEP+DDM (c) TEEP+IDA.

**Figure 12:**
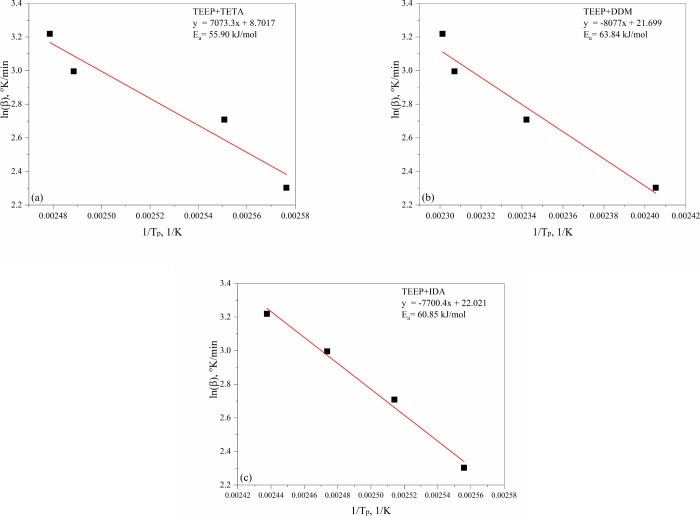
Plots of Ozawa evaluation for (a) TEEP+TETA (b) TEEP+DDM (c) TEEP+IDA.

Figures 11 and 12 present linear fit plots of the methods of Kissinger and Ozawa. Activation energies obtained from Kissinger and Ozawa methods are collected in Table 2. For the TEEP composition cured with triethylenetetramine, the activation energy values determined using the Kissinger and Ozawa methods were 65.38 and 55.90 kJ/mol, respectively. These values are similar to the DGEBA/TETA (e.g., 60.93 kJ) system described in other works.^51^ For TEEP systems with 4,4′-diaminodiphenylmethane (DDM), the activation energy values were at a similar level and amounted to 60.09 and 63.84 kJ/mol for the Kissinger and Ozawa tests, respectively. These values are similar to the diglycidyl ether of bisphenol A composition cured with amines of this type e.g. DGEBA/DDM (54 kJ/mol) and e.g. DGEBA/DDS (66–69 kJ/mol).^52−55^ In the case of TEEP composition cured with isophoronediamine, the activation energies were 57.36 and 60.85 kJ/mol in the Kissinger and Ozawa tests, respectively. The reported DGEBA/IDA systems in the literature showed Ea of 69 kJ/mol.^56^ The lowest activation energy (Ozawa model) of the cross-linking process was noted for the composition cured with TETA (55.90 kJ/mol), as well as IDA (60.85 kJ/mol). This may be due to the more complex structure of DDM leading to stronger steric restriction and thus attack on the oxirane ring than TETA and IDA.^57^ In addition, the low viscosity ensures good miscibility with the bioepoxy monomer, and the presence of a primary aliphatic group in these curing agents allows to obtain appropriate reactivity of the ingredients.

### Isoconversional Study

The standard epoxy resin curing reaction is an exothermic reaction between the epoxy groups and the active hydrogens of the curing agent to form a cross-linked material. The conversion can be expressed by the following eq 6, assuming that the heat of reaction generated during the curing reaction is directly proportional to the consumption of epoxy groups^58^
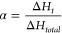
6

**Figure 13:**
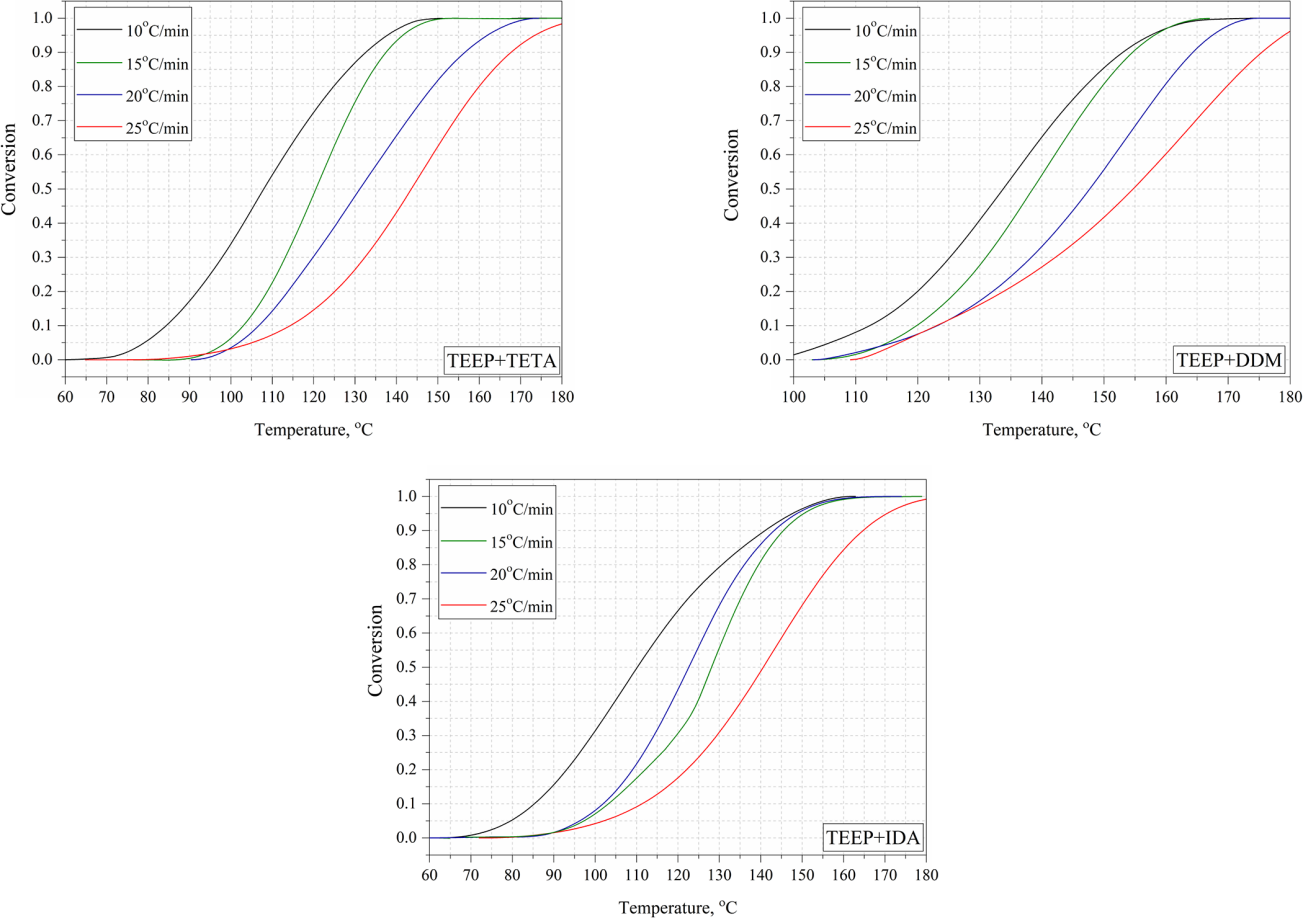
Chemical conversion data as a function of temperature calculated from dynamic DSC curves at different heating rates.

In eq 6, α is chemical conversion, Δ*H*t is the cumulative reaction heat from time 0 to time *t*, and Δ*H* total is the total heat of reaction for the complete conversion of 1. Chemical conversion data as a function of temperature calculated from dynamic DSC curves at different heating rates are shown in Figure 13.

The degree of conversion was dependent on the type of curing agent used. It can be seen that in the case of TETA and IDA curing agents, the degree of conversion 1 was achieved at lower temperatures than for DDM curing agents. From a processing point of view, it may be advantageous that the full cross-linking of the bioresin using TETA and IDA amines will not require as high a temperature as for DDM. On the other hand, DDM cured bioresin compositions will be able to be used for curing at elevated temperatures or quick pressing.

### Degradation Kinetics

Ozawa’s method is created on TGA curves recorded at various heating rates (β).^59^ It also takes in the degree of conversion α, which is defined as the ratio of the actual weight loss to the total weight loss, α = *m*_0_ – *m*/*m*_0_ – *m*_∞_, where *m* is the actual weight at time *t* (or at temperature *T*); *m*_0_ is the initial weight, and *m*_∞_ is the weight at the end of isothermal or nonisothermal experiments.^60^ As a result, the rate of degradation dα/dt, depends on the temperature and the weight of a sample, as presented by eq 7.
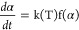
7Where: k(T) is the rate constant and f(α) is a function of conversion. If we assume that

k(T) = A exp(−*E*_a_/*RT*) and f(α) = (1−α)n, then eq 7 it can be described as present in eq 8:
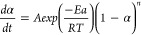
8Where: A is the pre-exponential factor; *E*_a_ is activation energy; R is gas constant; T is absolute temperature, and n is the reaction order.

Then we obtain eq 9 and based on the known conversion rate α and heating rate β, activation energy values Ea can be calculated.
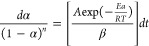
9

Based on eqs 7–(9) Ozawa’s method allows us to determine the activation energy from the plots of log β versus 1/T for a defined value of α. The energy of activation can be calculated from the slopes of the lines according to eq 10.
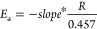
10

**Figure 14:**
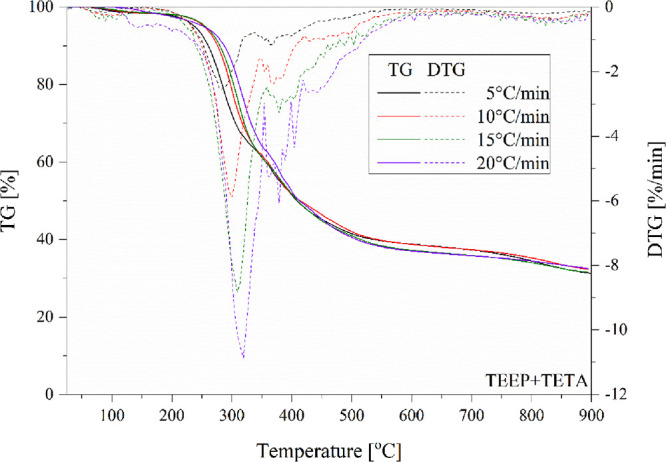
TGA and DTG curves of the TEEP with TETA recorded at different heating rates.

**Figure 15:**
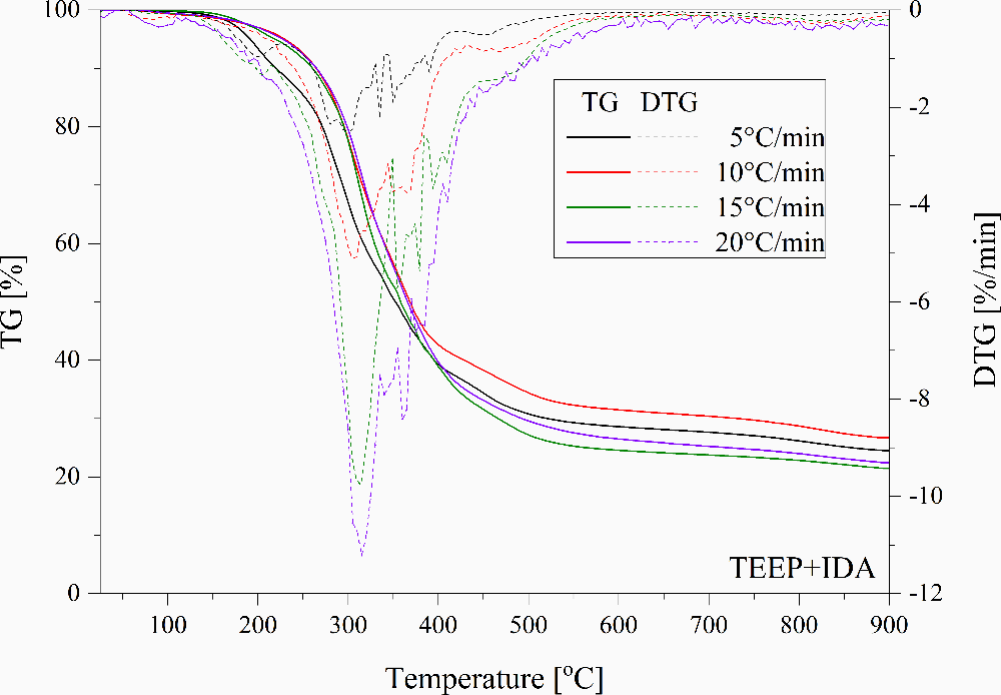
TGA and DTG curves of the TEEP with IDA recorded at different heating rates.

**Figure 16:**
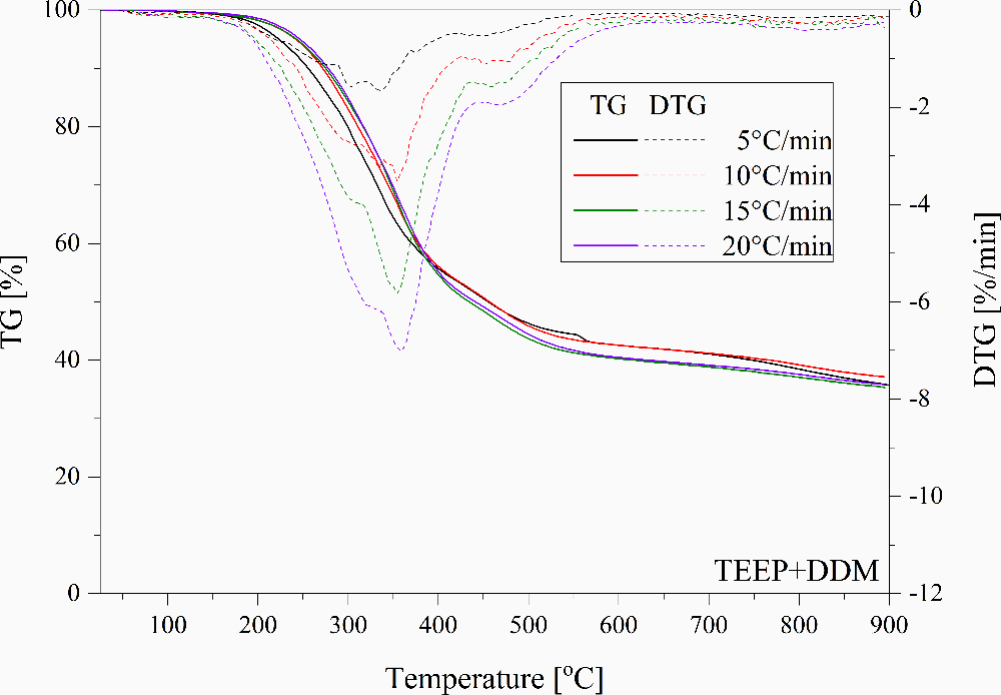
TGA and DTG curves of the TEEP with DDM recorded at different heating rates.

Table 3 summarizes the data obtained during nonisothermal TGA analyses and *E*_a_ values determined by the Ozawa method. Based on them, it can be concluded that all bioresin samples indicated the fastest decomposition at a temperature of approximately 300 °C. Moreover, based on the curves it can be seen that the carbonization process occurs between 400 to 500 °C (Figure 14–16). For all tested eugenol-based systems, the formation of quite a large amount of charred residue (approximately 25–35%) was observed, which will have a beneficial effect on the fire resistance of the tested materials.^61^ The introduction of phosphorus into the main chain of epoxy resin significantly improves thermal properties, which should translate into better fire resistance.^29^ The thermal degradation activation energy *E*_a_ (kJ/mol), with various conversions (cnv) by Ozawa’s method are collected in Table 4.

**Table 3:** TGA Data from Non-Isothermal Examinations

Name	β (°C/min)	T10% (°C)	Residual Mass (%)	DTG Peak Temperature (°C)	Max Degradation Rate (%/min)	Ozawa *E*_a_ (kJ/mol) (α=0.5)
TEEP+TETA	5	260.8	31.2	281.6	2.6	211.8
	10	276.5	32.2	298.8	5.9	
	15	280.1	31.5	310.1	8.8	
	20	287.5	32.5	320.3	11.0	
TEEP+DDM	5	287.2	35.5	330.8	3.9	222.3
	10	271.7	37.1	357.2	3.6	
	15	274.9	35.3	355.8	5.8	
	20	278.0	35.8	360.1	7.1	
TEEP+IDA	5	219.8	24.4	295.9	2.6	199.4
	10	264.6	26.6	306.3	5.2	
	15	260.5	21,4	314.4	9.8	
	20	264.8	22.4	314.8	11.2	

**Table 4:** Thermal Degradation Activation Energy *E*_a_ (kJ/mol), with Various Conversions (cnv) by Ozawa’s Method

	TEEP+TETA	TEEP+DDM	TEEP+IDA
cnv	*E*_*a*_ (kJ/mol)	*E*_*a*_ (kJ/mol)	*E*_*a*_ (kJ/mol)
0.1	159.9	357.3	58.4
0.2	134.9	178.3	108.3
0.3	132.1	260.6	116.2
0.4	178.2	270.2	189.7
0.5	211.8	222.3	199.4
0.6	402.8	311.4	143.6
0.7	744.9	577.2	356.4
0.8	216.9	420.1	401.7

The values of the activation energy *E*_a_ of the thermal degradation process increase simultaneously with the degree of conversion 0.3 to 0.7, especially for samples TEEP + TETA and 0.2 to 0.5 for TEEP+IDA. The highest Ea value among the tested materials at degree 0.5 was recorded for the TEEP+DDM composition (222.3 kJ/mol). This is mainly due to the structure of the hardener used and its reactivity with the epoxy system. The presence of an aromatic ring in the structure of the curing agent resulted in improved thermostability and the highest char yield.^62^

Comparison of TGA analysis results for TEEP samples and commercial resins tested in nitrogen and air atmospheres are summarized in Table 5. It can be seen that TEEP-based resins begin to decompose in nitrogen from 264 to 276 °C and in air from 207 to 298 °C. In a much lower temperature range than DGEBA-based resins. For which the beginning of degradation falls in the range 260–387 °C in nitrogen and 327–385 °C in air. It should be emphasized that, compared to commercial resins, the TEEP bioresin has a much lower maximum degradation rate determined by DTG and a higher amount of burnout residue after thermal degradation, both in nitrogen and in air. It should be emphasized that an increase in the amount of chars may reduce the production of flammable gases, reduce the exothermicity of the pyrolysis reaction, and reduce the thermal conductivity.^63^ This phenomenon will have a beneficial effect on the thermal resistance of these materials, determining their area of use as materials with increased fire resistance. The improved thermal stability of bioepoxy resin enables wider use of this type of material in areas such as construction, automotive or the production of high-strength adhesives and coatings, where fire safety and stability of properties under various temperature conditions are required.^24^

**Table 5:** Comparison of TGA analysis results for TEEP samples and commercial resins tested in nitrogen and air atmospheres

	T10% (°C)	Residual Mass (%)	DTG Peak (°C)/max.rate (%/min)	T10% (°C)	Residual Mass (%)	DTG Peak (°C)/ max. rate (%/min)
Name	nitrogen			air		
TEEP+TETA	276.5	32.2	298.8/5.9	268.8	7.47	341.9/5.22
TEEP+DDM	271.7	37.1	357.2/3.6	298.6	7.77	329.1/4.92
TEEP+IDA	264.6	26.6	306.3/5.2	207.2	7.04	317.4/7.37
DGEBA+ TETA	353.4	6.99	374.6/20.74	355.0	0	366.3/13.01
DGEBA+ DDM	387.0	15.17	390.4/22.27	385.1	0	389.2/18.95
DGEBA+ IDA	260.6	5.98	370.8/14.67	368.9	0	368.9/7.59
GEDEO	328.3	4.16	328.7/22.21	327.5	0	327.5/19.8

## Conclusions

In this work, an epoxy biomonomer was successfully synthesized from eugenol. The structure of the obtained compound was confirmed by NMR analysis. The resulting liquid monomer was cured using three types of amine curing agents. The cross-linking process was monitored using FTIR and nonisothermal DSC analysis. According to the Kissinger and Ozawa method, the activation energies of this process were determined, which enabled the assessment of the efficiency of the tested curing systems. The calculated activation energies according to Kissinger and Ozawa were 65.38 and 55.90 kJ/mol for TEEP+TETA; 60.09 and 63.84 kJ/mol for TEEP+DDM, 57.36 and 60.85 kJ/mol for TEEP+IDA, respectively. The lowest activation energy of the cross-linking process was recorded for the composition cured with isophorone diamine as well as triethylenetetramine, which may be mainly due to good miscibility and low viscosity of this curing agents. In addition, the degradation kinetics of the cured epoxy materials were assessed using nonisothermal TGA analysis according to the Ozawa method. It should be emphasized that the presented characteristics bring a lot of important data to classify a new type of epoxy bioresin and determine its future area of application. It should be highlighted that an increase in the amount of burnout char residue for investigated bioresin may reduce the production of flammable gases and improve the fire resistance of the materials. The obtained research results confirmed that phosphorus-modified bioresin produced on the basis of eugenol has favorable thermal properties, allowing its use in, among others, fire-resistant coatings or sustainable building materials. The knowledge based on the presented kinetic data can be used in industrial processes, enabling the control of properties and appropriate processing parameters of the new type of bioresin produced from eugenol.

## Data Availability

The data that support the findings of this study are available from the corresponding author (Danuta Matykiewicz) on request and in the data set: https://doi.org/10.18150/BGX9CO
